# Magnetic tunneling with CNT-based metamaterial

**DOI:** 10.1038/s41598-019-39325-9

**Published:** 2019-02-22

**Authors:** Gunther Kletetschka, Yoku Inoue, Jan Lindauer, Zdeněk Hůlka

**Affiliations:** 1 Institute of Geology, Czech Academy of Sciences, Rozvojova 269, Prague, Czech Republic; 2 Department of Applied Geophysics, Charles Univ, Albertov 6, Prague, Czech Republic; 3 Geophysical Institute, University of Alaska Fairbanks, 903 N Koyukuk Drive, Fairbanks, AK USA; 4 Department of Electronics and Materials Science, Shizuoka University, 3-5-1 Johoku, Naka-ku, Hamamatsu, 432-8561 Japan; 5 High School (Gymnazium), Karlovy Vary, Czech Republic; 6 ZH Instruments Inc., Brno, Czech Republic

## Abstract

Multiwall carbon nanotubes (MWCNTs) fabricated by chemical vapor deposition contain magnetic nanoparticles. While increasing frequency of electromagnetic field (EMF) exposure (up to <10 kHz) of MWCNTs resulted in slight induced magnetization decrease due to skin effect of the conducting carbon, we discovered that higher frequencies (>10 kHz) contained an exponential magnetization increase. We show that puzzling magnetization increase with decreasing magnetic field amplitude (less than 0.5 A/m for 512 kHz) is due to matching the field amplitudes of the magnetic nanoparticles inside nanotubes. This observation reveals a possibility of magnetic tunneling in MWCNTs (change of magnetic state of blocked magnetic moments). This interpretation is supported by observation of unblocking larger magnetic remanence (MR) portion from MWCNTs with progressively smaller amplitude of oscillating magnetic field.

## Introduction

**Figure 1:**
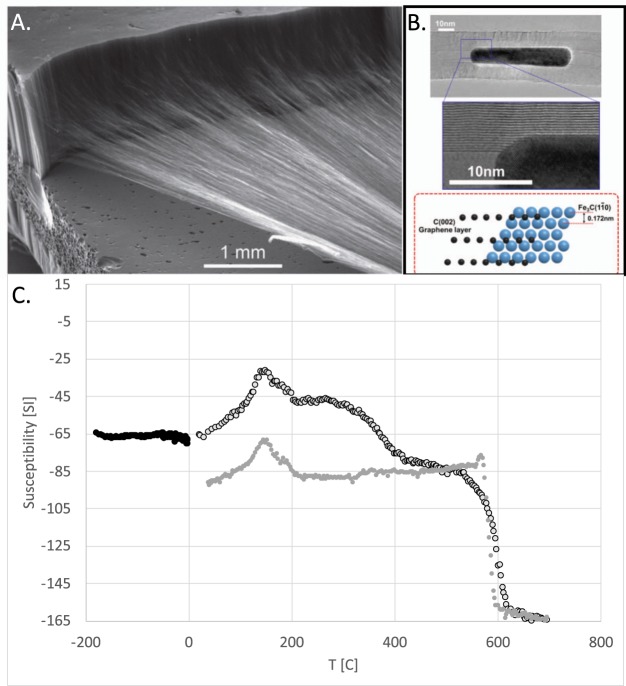
Multiwall Carbon Nanotubes (MWCNTs); (**a**) Drawing continuous field of filaments from vertically aligned MWCNTs. (**b**) Nature of the contact between MWCNTs and iron rich nanoparticles. Fe_3_C structures are visible inside MWCNTs. Lattice match relates to C(002) ~ 2xFe_3_C(110). (**c**) Magnetic susceptibility of MWCNTs showing 4 drops in induced magnetization, at 230 C, 400 C, 580 C, Hematite 700 C.

**Figure 2:**
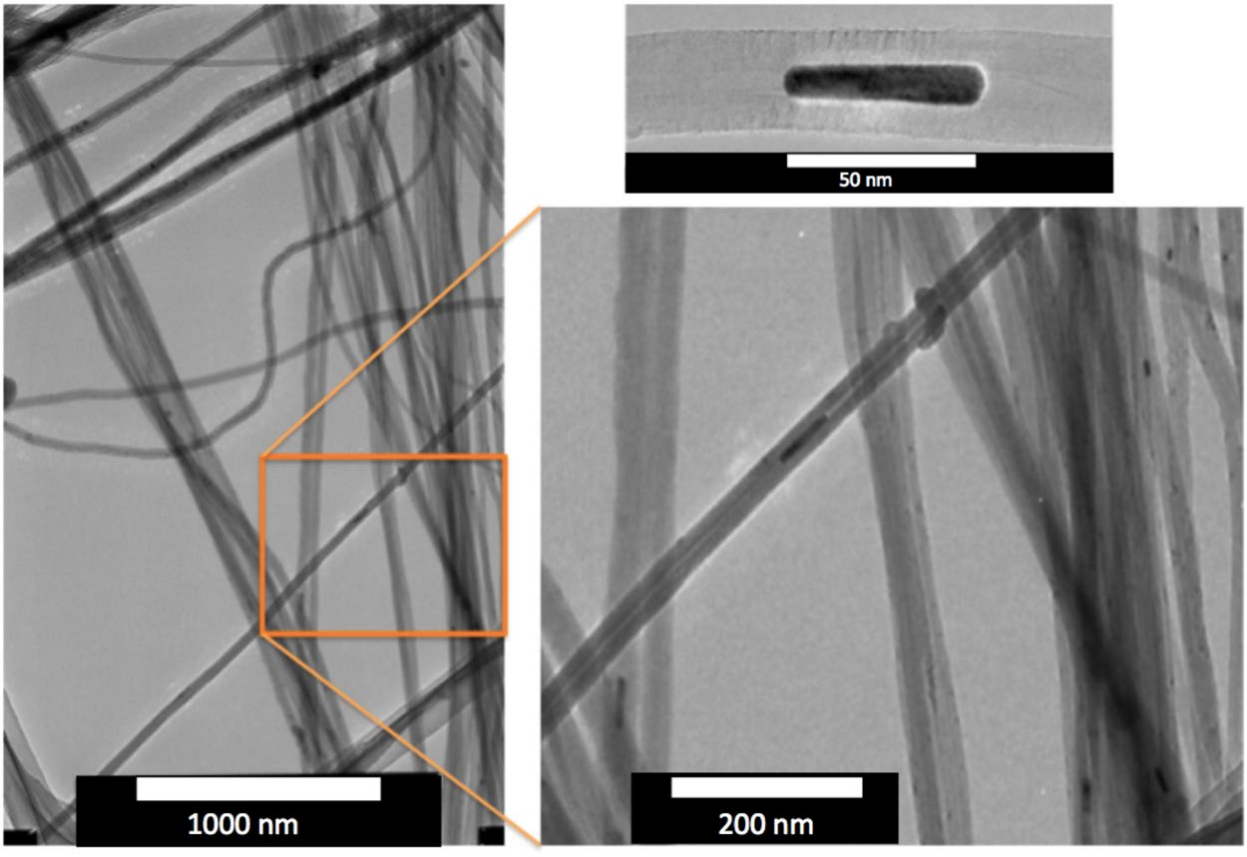
Multiwall Carbon Nanotubes contain nanoparticles of iron oxide inside them shown in darker gray color shade. Image on the right side is an enlarged section showing 50 nm × 5 nm iron oxide grain. Upper right image shows another detail of the iron oxide grain inside the multiwall carbon nanotube.

Carbon nanotubes became widely produced product due to broad range of applicability of this material. This material has been used for microwave absorption properties when combining them externally with iron oxide nanoparticles^1^. Spinnable multiwall carbon nanotubes (MWCNTs) contain multiple (up to 100) conducting graphene walls and provide an unusual conducting structure that may be utilized in development of new materials. We chose this material because of an unknown and unresearched response of these MWCNT structures to high frequency oscillating magnetic fields. Such structures were fabricated with chemical vapor deposition^2,3^ assisted with iron chloride vapor as a nucleation agent (Fig. 1). Observation in transmission electron microscope revealed that CNTs have nanosized iron rich particles (at most 3 wt%) inside them (Fig. 2). The amount of iron in the MWCNT sample was measured by thermogravimetric analysis (TGA) technique^4^. The present results were taken from one sample and thus we assume that all our results are based on the same weight of the material. These ferromagnetic particles interact with MWCNTs and form a new material with unique electromagnetic properties^5^.

Nanosized magnetic grains at room temperature (20–25 °C) are at superparamagnetic (SP) state^6,7^. For magnetite the size of such particle has diameter <20 nm while for iron this maximum diameter is less than 10 nm. The smaller size for iron has to do with its larger saturation magnetization^8–10^. SP nanoparticles (SPNs) have unstable magnetic moments at room temperature, fluctuating in random directions. In SP-state each magnetic grain contains a single magnetic domain (macrospin = SD). Due to the inherent magnetic anisotropy (crystalline, shape, stress due to lattice of surrounding minerals) magnetic direction is residing in two stable orientations (parallel and antiparallel to the so called easy axis of mineral) separated by an energy barrier. This barrier depends on the degree of anisotropy. Given a specific temperature T, the associated lattice vibration is tied with the probability that the direction of the magnetic domain can be flipped from parallel to antiparallel and vice versa. We define the mean time *t* between the two flips according to the Neel-Arrhenius equation^11^:

1 $$t={t}_{0}{e}^{(\frac{KV}{{k}_{B}T})}$$

where *t*_0_ is attempt time and depends on the mineral composition (typical value is hundreds of picoseconds), *K* is magnetic anisotropy level, *V* is volume of the SPN, and *k*_*B*_ is the Boltzmann constant (1.3806488 × 10^−23^ J/K). Once the *t* becomes progressively larger than specific laboratory time scale observation (e. g. minutes) the magnetic moment is blocked for that time period and the probability of crossing the barrier from one magnetic state to the other becomes increasingly lower.

MWCNTs contain iron/iron oxide nanoparticles whose portion would behave as ferromagnets in SP state, if not part of MWCNTs. These nanoparticles are, however, surrounded by electrically conducting graphene tubes and this conductivity interferes with the magnetic moment blocking of nanoparticles that would otherwise behave as SPN. Tight arrangement between SPN and CNTs defines a new type of metamaterial. In nature SPNs are constantly activated by phonons. However, manmade tight graphene layers around the SPN would force SPNs to behave like SD grains.

In this work we demonstrate functionality of this new metamaterial system of conducting tubes with SPNs by electromagnetic tests. We exposed MWCNTs to frequency and amplitude dependent electromagnetic fields (EMFs). We used a hollowed electric coil oscillating in various frequencies and strengths (SM100/105-ZH Instruments Inc.) and monitored the material’s induced magnetization. Then we used superconducting coil system (2G Enterprises Inc.) and monitored material’s magnetic remanence (MR).

## Results

### Exposure of carbon nanotubes to EMF

**Figure 3:**
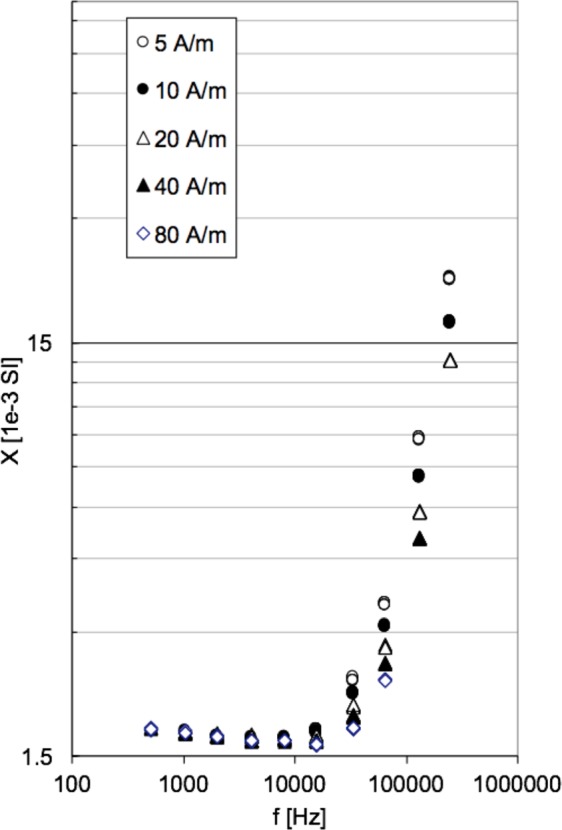
Magnetic susceptibility of the multiwall carbon nanotubes (MWCNTs). Contrasting symbols refer to the amplitude of the magnetic field of given frequency (256 kHz max) applied to MWCNTs.

**Figure 4:**
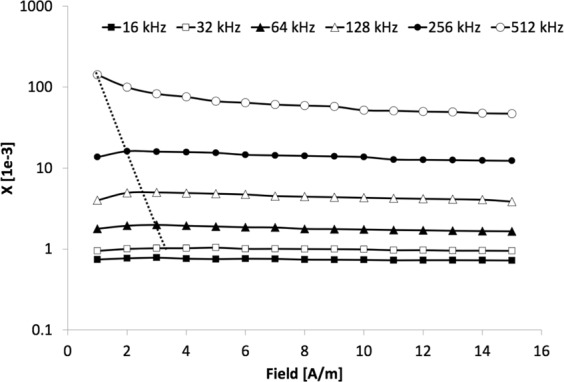
High frequency (16 to 512 kHz) magnetic susceptibility of the multiwall carbon nanotubes (MWCNTs) in fields between (1 to 15 A/m). Symbols/lines refer to the frequency of the magnetic field of given field amplitude applied to MWCNTs. Dotted line indicates approximate variation of the maximum magnetic susceptibility for separate frequencies.

MWCNTs (net weight 2.022 g) inside plastic cylindrical (4 cm × 2.5 cm radius) container was exposed to variation of EMFs (Fig. 3). MWCNTs’ induced magnetization (reflected by magnetic susceptibility) decreased with increased EMFs’ frequency until about 10 kHz. For this frequency range, there was not significant difference in susceptibility in respect to EMFs amplitude. EMFs with >10 kHz, however, showed exponential susceptibility increase that continued beyond 512 KHz (Fig. 4). Furthermore, this EMFs’ frequency range showed an inverse proportionality of the susceptibility with applied EMFs’ amplitude. This trend was verified in different ZH Instruments’ laboratory in Brno, where it further increased for the EMFs smaller in amplitude (<5 A/m) and higher frequency (512 kHz). Figure 4 also showed that frequencies less than 512 KHz had induced magnetization maximum between 2 and 4 A/m, while for 512 KHz, the trend suggested that the maximum susceptibility is achieved for EMFs smaller in amplitude than 0.5 A/m.

### Effect of magnetic remanence of nanotubes when exposed to EMF

**Figure 5:**
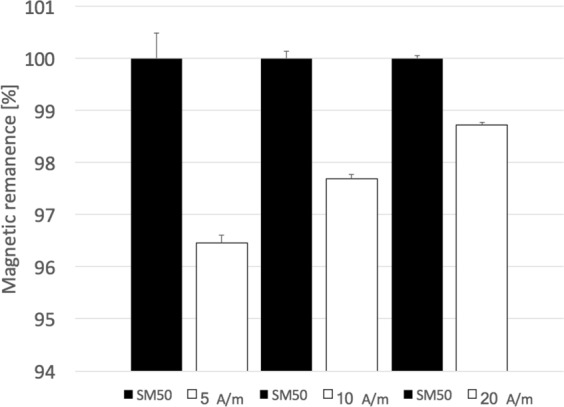
MWCNT’s magnetic remanence acquired by 144,000 A/m direct current pulse and subsequently demagnetized with 40000 A/m alternating field (SM50) with frequency 150 Hz is shown in comparison after exposing the same sample to the fields 5 A/m, 10 A/m, and 20 A/m, all with frequency of 250,000 Hz. Error bars show standard deviation from 3 measurements.

Figure 5 illustrated how the exposure to EMFs with various frequencies changed MWCNTs’ MR. MWCNTs’ exposure to EMF of 250 MHz and variable field amplitude resulted in MR decrease in general. Importantly, the 2-min exposure to smaller field amplitude (5 A/m) resulted in larger MR lost (3.5%) than 2-minute exposure to the field of 20 A/m that resulted in loss of <1.5% of MR. Intermediate field 10 A/m had 2.5% loss of MR.

## Discussion

Unique fabrication of MWCNTs forest^2,3^ allowed production of iron-rich nanoparticles as a by-product. While we did not attempt to find the controlling variable for the particle size and count, the resulting observed particles were of cylindrical shape filling parts of the internal space of the MWCNTs (Fig. 2). We found that the interface between graphene material and magnetic nanoparticle contains Fe_3_C in addition to magnetite, maghemite, and hematite. CNT growth is parallel with a mixture of Fe_3_C particle, gamma Fe_2_O_3_, alfa Fe_2_O_3_ and Fe_3_O_4_. For this we have evidence in Fig. 1, where the temperature dependent magnetic susceptibility indicated transition temperatures near 230 C, 400 C, 580 C, and above 580 C, indicating the presence of cementite (Fe_3_C), maghemite (gamma Fe2O3), magnetite (Fe_3_O_4_), and hematite (Fe_3_O_4_) [*Cheng et al*., 2014; *Smith et al*., 1911]. For MWCNT fabrication cementite plays a role. After exceeding solubility limit of carbon in iron, which is Fe_3_C, CNT gets to precipitate from the particle [*Odunmbaku et al*., 2019].

Presence of these SPNs displayed unique magnetic phenomenon that was not seen in natural materials before. The specific MWCNTs containing SPNs reflects unusual properties of exponential increase of its magnetic susceptibility with frequency and, as such, should be classified as metamaterial^12,13^. EMFs with frequency below 10 kHz caused the susceptibility to slightly decrease with EMFs’ frequency (Fig. 3). This phenomenon is trivial due to a conducting nature of MWCNTs. Conducting magnetic material generates electric currents that are arranged so that they lower the external change of magnetic field. With increasing frequency, the current induced in conducting material per unit of time is larger but shifted by pi/2 in respect to oscillating frequency of magnetic field. So, when the ambient field is changing from one sign to the other, the inducing current is maximum and generates magnetic field in opposing direction. When the frequency is low, the currents decay due to ohmic losses and the ambient field penetrates towards the nanoparticles inside the tubes. So, for low frequency of ambient field, the induced currents decay to zero within the oscillation period and thus the magnetic grains can react to the ambient field by magnetizing in the same direction and greater magnetic susceptibility for lower frequencies. When the EMFs’ frequency increases, the induced currents do not decay entirely within the cycle and contribute with magnetic field component in opposing direction than ambient magnetic field. This results in overall magnetic susceptibility decrease for frequencies less than 10 kHz as seen in Fig. 3. This feature should continue also for frequencies larger than 10 kHz but Fig. 3 indicates that there is another more dominant effect that takes place.

For EMFs with >10 kHz the magnetic susceptibility reversed its trend and exponentially increased with frequency. We interpret this behavior as due to an increased match between EMF’s frequency and frequency of the microcurrents from SPN’s. SPNs contain their macromoments with frequency spectra that are controlled by SPNs’ shape, mineralogy, and internal magnetic coercivity^9,14^. We hypothesize that once the SPN’s frequency spectra from macromoments begins to generate microcurrents that overlap with the EMFs from the applied coil, both fields begin to resonate and cancel each other and the extra released energy allows an unblocking the SD SPNs so they can flip between the two states of magnetizations near the resonant frequency. This is an analogy of the tunneling between quantum states and for the purpose of this paper we call it “magnetic tunneling”.

Technically, MWCNT metamaterial prohibits this kind of moment fluctuation of SPN due to conducting nature of surrounding graphene material. Magnetic moments are locked in the conducting graphene structure and behave like single domain states. The phonon energy is not large enough to allow magnetic transition despite the small grain size and low remanence blocking temperature of such material. Despite the blocking, the macromoments’ oscillation generates spectrum of micro-currents in the surrounding MWCNT material and frequency of these microcurrents can overlap with the ambient coil EMF frequencies. MWCNTs contain magnetic grains of various sizes (Fig. 2) and these would generate wide macrocurrents’ EMF frequency spectrum from 10^10^ Hz to 10^−10^ Hz^9,11^. It is reasonable to assume that the high EMF frequency of the ambient coil would start to significantly overlap the microcurrents’ generated by magnetic macromoments, and the magnetization block would be released from being trapped and would start to follow the external field direction, contributing to the overall magnetic susceptibility.

The efficiency of unblocking the SPN state inside MWCNTs would increase with the match of the microamplitudes of the currents from individual nanoparticles. Thus, the smaller the applied current the more effective would be the overlap with the microcurrent spectrum of specific SPN range and SD unblocking.

In order to test if these hypotheses were valid, we show that the metamaterial system would be losing magnetic remanence when exposing to the low amplitude and high frequency magnetic fields. Figure 5 provides a demonstration that portion of magnetic remanence is lost upon EMF amplitude exposure. Furthermore, Fig. 5 indicates that with the smaller the amplitude of the exposing field, the larger the amount of magnetic remanence is being lost due to unblocking the SP nature of the trapper SPN in SD magnetic state. This can be illustrated by the following example: An electromagnetic field exposure (EMF) of our material to 5 A/m and 250 kHz caused ~3.60% loss of magnetic remanence. However, the EMF exposure to 20 A/m and 250 kHz caused only ~1.25% loss in magnetic remanence (see Fig. 5).

We fabricated a new type of metamaterial that is capable to block the magnetic moments of SPNs forcing them to behave like single domain magnetic states despite the nanosize volume and room temperature (20–25 °C).

We demonstrated that the blocked SPN can be unblocked by physical analogy to tunneling, when we provide the necessary magnetic oscillation resonant energy that allows to unblock SD moments so they can flip between their stable magnetic domain states.

## Methods

### Fabrication of nanotubes

MWCNTs were prepared by chemical vapor deposition process on silicon wafer. Flowing acetylene into a vacuum furnace is the main fabrication principle. In front of the maximum heat location (820 C) is a variable amount of iron chloride. Iron chloride vaporizes at temperature between 500 and 600 C. The vapor reacts with acetylene, chloride atoms bind with hydrogen atoms that are part of acetylene. Remaining iron forms tiny pools of liquid Fe-Cl solution. Pools precipitate and create a nucleation sites for MWCNT forest. The carbon released from acetylene becomes dissolved in the liquid pools and supplies the material for building MWCNT forest (Fig. 1). Some of the tops of the catalyst site are lifted up with growing MWCNTs and then nucleates and form small particles inside the MWCNT forest (Fig. 2).

### EMF exposure of nanotubes

1 mm long forest of MWCNTs were put inside the plastic container of cylindrical shape, (2.5 cm diameter × 4 cm long) to have its net weight of 2.022 g. Then sample was periodically exposed to variable field frequency and amplitude inside the coil of an instrument SM105 (ZH Instruments Inc.) designed to measure frequency dependent magnetic susceptibility. For magnetic remanence measurement we used superconducting rock magnetometer (2 G Enterprices Inc.).

Sample of MWCNTs (2.022 g) were exposed to pulse magnetic field of 144,000 A/m and subsequently demagnetized with 40000 A/m alternating field (SM50) with frequency 150 Hz. Sample was for 2 minutes exposed to the alternating fields 5 A/m, 10 A/m, and 20 A/m, all with frequency of 250,000 Hz. Error bars showed standard deviation from 3 measurements (Fig. 5).

The material will be provided upon request. The data and associated protocols will be available at the time of publication on public domain web site.

## Supplementary information

Supplementary information

## Data Availability

All materials, data and associated protocols can become available upon request.
