# Supplementary material for: Magnetic tunneling with CNT-based metamaterial
Source: Sci Rep. 2019 Feb 22;9:2551. doi: 10.1038/s41598-019-39325-9 (PMC6385282; doi:10.1038/s41598-019-39325-9)
Supplement: Supplementary file 1 — Supplementary information [file 41598_2019_39325_MOESM1_ESM.pdf]

Supplementary material

Magnetic tunneling with CNT-based metamaterial

Gunther Kletetschka[1,2,3], Yoku Inoue[4], Jan Lindauer[5], and Zdenek Hulka[6]

[1]Institute of Geology, Czech Academy of Sciences, Rozvojova 269, Prague, Czech Republic

[2]Department of Applied Geophysics, Charles Univ, Albertov 6, Prague, Czech Republic

[3]Geophysical Institute, University of Alaska Fairbanks, 903 N Koyukuk Drive, Fairbanks, AK, USA

[4]Department of Electronics and Materials Science, Shizuoka University, 3-5-1 Johoku, Naka-ku, Hamamatsu 432-8561, Japan

[5]High School (Gymnazium) Karlovy Vary, Czech Republic

[6]ZH Instruments Inc. Brno, Czech Republic

Figure S1 shows a typical hysteresis loop of the MWCNT material. Narrowing of the hysteresis near the center indicates a presence of both the superparamagnetic and single domain state magnetic grains(Tauxe et al., 1996).

Figure S2 shows proportionality of magnetic susceptibility with the mass of the MWCNT. Magnetic susceptibility decreases in both cases as a result of increased thermal energy that randomizes the induced magnetization level (Dunlop and Özdemir, 1997; Kletetschka and Wieczorek, 2017).

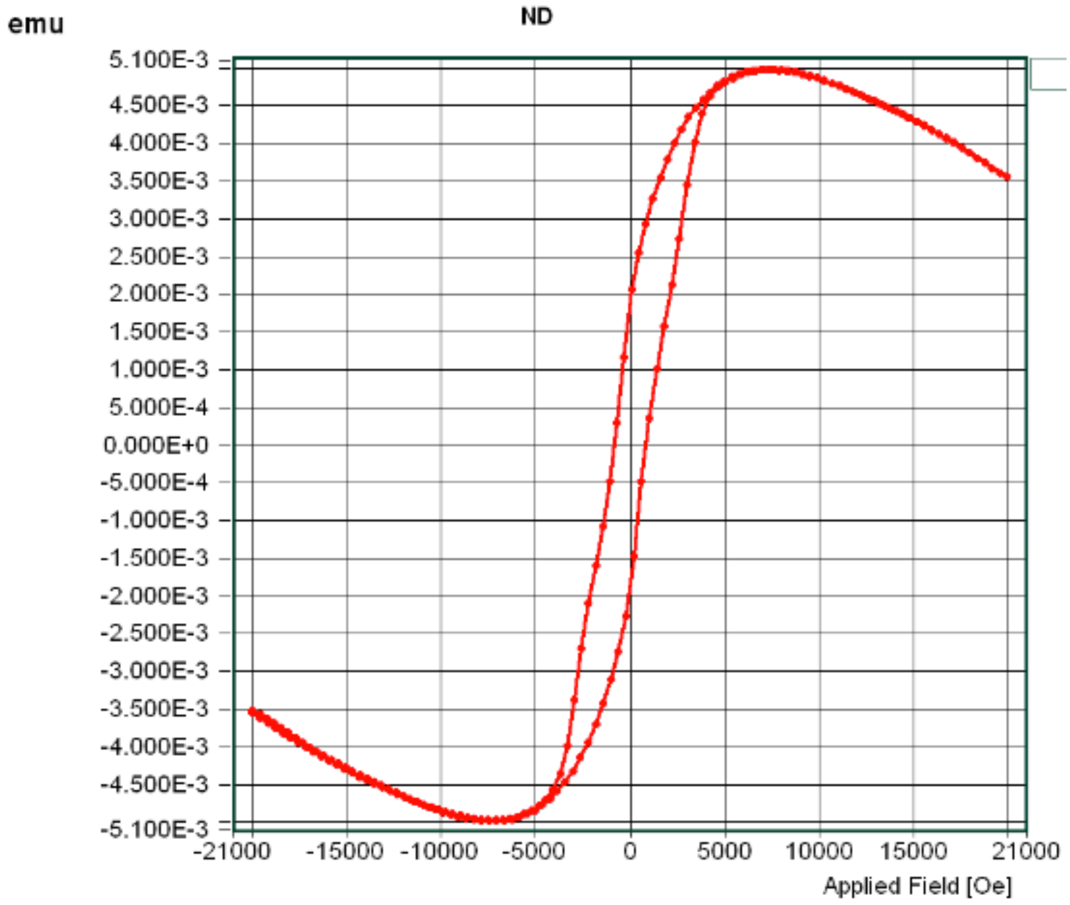

Figure S1: Hysteresis loop of MWCNTs

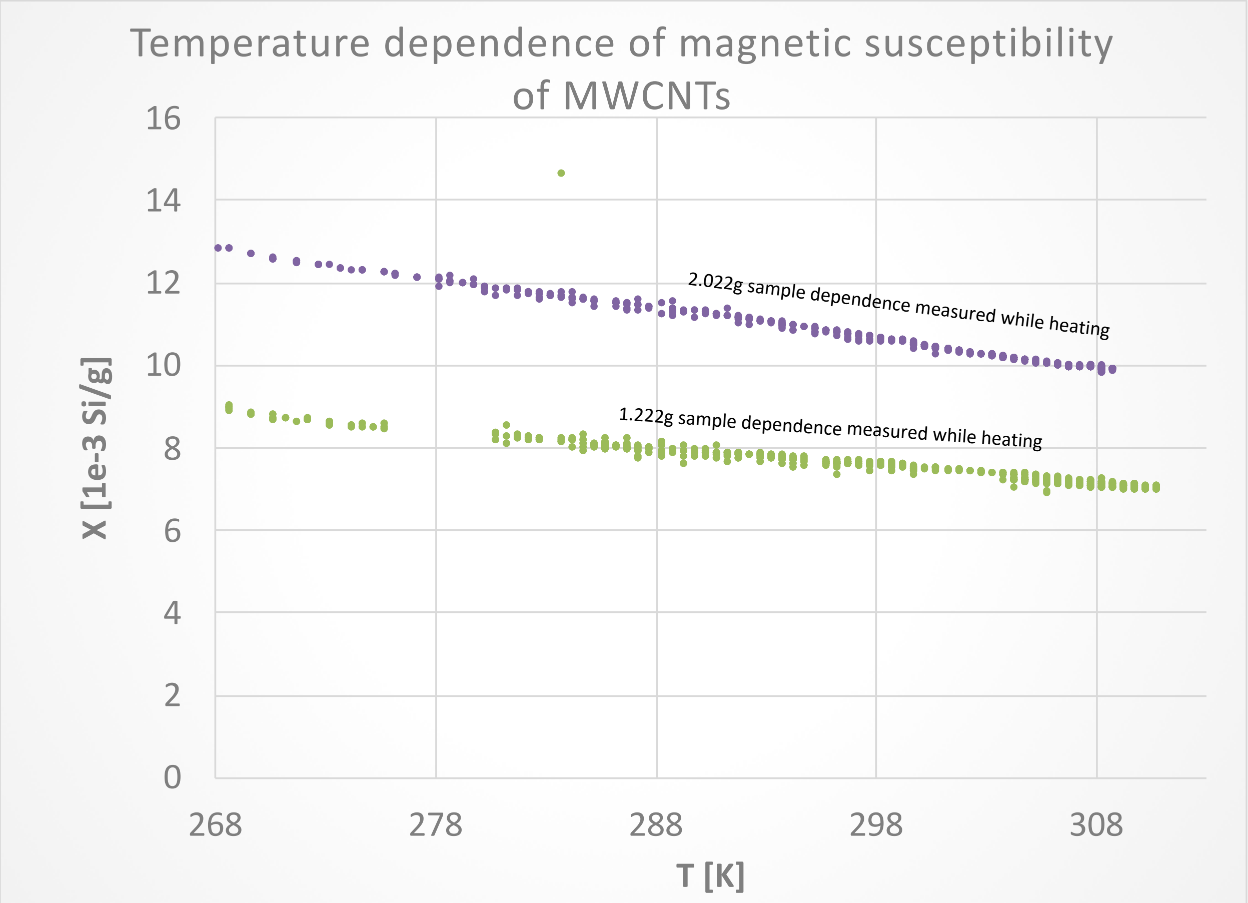

Figure S2:
Weight dependence of magnetic susceptibility on temperature near the room temperature range (268-308 K) when using exposure to 256 KHz and 5 A/m electromagnetic field.

Reference:

Dunlop, D.D., Özdemir, Ö., 1997. Rock Magnetism: Fundamental and Frontiers. Cambridge University Press, Cambridge.

Kletetschka, G., Wieczorek, M.A., 2017. Fundamental Relations of Mineral Specific Magnetic Carriers for Paleointensity Determination. Physics of the Earth and Planetary Interiors 272, 44-49.

Tauxe, L., Mullender, T.A.T., Pick, T., 1996. Potbellies, wasp-waists, and superparamagnetism in magnetic hysteresis. J. Geophys. Res.-Solid Earth 101, 571-583.
